# NETosis

**DOI:** 10.1155/2013/651497

**Published:** 2013-02-05

**Authors:** Miguel Antonio Mesa, Gloria Vasquez

**Affiliations:** Rheumatology Section, Universidad de Antioquia, Medellin, Colombia

## Abstract

Neutrophils are the first line of defense of the immune system against infection. Among their weaponry, they have the ability to mix and extrude their DNA and bactericidal molecules creating NET-like structures in a unique type of cell death called NETosis. This process is important in order to control extracellular infections limiting collateral damage. Its aberrant function has been implicated in several human diseases including sepsis and autoimmune disease. The purpose of the present paper is to give a general introduction to this concept.

## 1. (N)ETosis

Polisegmented granular cells are the immune system's first line of defense against infections. They have been considered short-lived cells with unspecific functions of phagocytosis, granular secretion, and the production of reactive oxygen species (ROS) [1]. This knowledge has been challenged by several lines of evidence, including the formation of extracellular traps (ET), a defense mechanism consisting of the extrusion of intracellular material in the form of neutrophils extracellular traps (NETs) to the surrounding extracellular medium in order to concentrate antibacterial substances and snare invading microorganisms; such a process is often accompanied by cell death. The purpose of the present paper is to give a general introduction to this concept.

## 2. The Neutrophil

Human neutrophils constitute the first line of defense of innate cellular immunity. As the most abundant subtype of leucocytes in peripheral blood, they constitute approximately 70% of these cells [2, 3]. They are terminally differentiated cells with a life span of 12 to 15 hours [4], whom after this time period undergo apoptosis: this life span can be extended after exposition to several substances like cytokines [5]. Under light microscopy, these cells have an approximate diameter of 12 to 15 *μ*m and a nucleus with several lobules [2]. They also have a cytoplasm rich in different granules plenty of antimicrobial peptides [3] and enzymes necessary to synthesize several substances, including arachidonic acid derivates with either inflammatory properties like tromboxans and leucotriens [6], or negative regulators of inflammation such as prostaglandins, lipoxins, protectins, and so forth. These substances can be produced entirely within the neutrophils [6] or in conjunction with other cells using transcellular pathways to produce lipoxins [7]. Neutrophils can produce chemokines, cytokines from the tumor necrosis factor (TNF) superfamily, angiogenic and fibrogenic factors, and pattern recognition molecules such as pentraxins, collectins, and ficolins [1]. Additionally, they have an enzymatic complex known as nicotinamide adenine dinucleotide phosphate (NADPH) oxidase type 2 (NOX2) responsible for the production of reactive oxygen species during the respiratory burst [8].

## 3. Cell Death

Despite the advances in the field of cell death since the description of apoptosis [14] in 1972, the determination of cell death and the distinct pathways that lead to it continue to be a matter of debate. Hence, in 2005, the nomenclature committee on cell death published a consensus on its classification [15]. This consensus was updated in 2009 [16] and in 2012 [17]. A cell can be classified as dead when it has lost its individuality or when it has reached a “point of no return” in which the cell loses its function permanently (Table 1). Given the growing body of evidence, the committee decided to change the classification system from the classical morphological classification to a biochemical one, allowing it to identify fourteen death models. Some of these models, nonetheless, are cell processes and only in certain circumstances become a cell death pathway, for example, autophagy [16]. In fact, autophagy prevents cellular senescence and provides energy during cellular starvation [18]. Additionally, autophagy is able to become part of other cell death subroutines in order to allow its proper execution (e.g., apoptosis and NETosis) [17, 19]. 

Although neutrophils die by apoptosis under physiological conditions [44], after their activation, they are able to switch to different types of cell death like autophagy [45] or NETosis [4, 44]. In the 2012 classification, NETosis is accepted for the first time as a specific cell death subroutine of granulocyte cells different from apoptosis and necrosis, based on the demonstration of its insensitivity to caspase inhibition and necrostatin, respectively. In the consensus, they note how NETosis shares characteristics and possibly enzymatic machinery with autophagy and regulated necrosis, concluding that currently it is not possible to define whether NETosis is a specific case of either or an independent cell death mechanism per se.

## 4. NETs Estructure

In 2004, Brinkmann et al. [9] described how after stimulation with interleukin (IL) 8, phorbol myristate acetate (PMA), or lipopolysaccharide (LPS) followed by dual visualization with electronic microscopy (EM) and immunofluorecence (IF), neutrophils formed previously unknown NET-like structures [9], which were named neutrophil extracellular traps (NETs). When observed under EM, these NETs were composed of linear elements about 15–17 nanometers (nm) in diameter and are studded with globules with a diameter of 25 nm and several of the former form broader elements of 50 nm with various substances grouped around this scaffold, mainly neutrophil granules proteins (Table 2) [10]. The backbone of these NETs was composed of deoxyribonucleic acid (DNA) and histones as demonstrated by IF, the resistance of NETs to degradation by proteases, and their destruction upon the use of DNA degrading enzymes [9]. 

These NETs showed to be delicate structures [46], requiring careful manipulation during the different steps of their isolation, activation, and visualization, where EM and IF are both required [9, 46], since fibrin can simulate NETs [30] in *in vivo* models, leading to confusion.

## 5. NETosis Is Found to Be a Type of Cell Death

In the original description of NET production [9], the authors considered NETosis was an active process not related to cell death based on several observations as follows.

- Stimuli that induce NETs did not promote the release of cytoplasmic markers like lactate dehydrogenase, and activated cells excluded vital dyes for at least two hours after stimulation, ruling out necrosis as an associated phenomenon.
- IL-8 and LPS which usually prolong neutrophil life span-induced NET formation.
- NETs were formed by motile cells as evidenced through time-lapse video microscopy.
- NETs were formed as early as ten minutes after activation—a time course not compatible with apoptosis.

This hypothesis was received with disbelief and it was considered that a process that leads to nuclear disintegration with DNA extrusion into the extracellular space should inevitably lead to cell death, as evidenced in an editorial written by Dr. Lee in 2004 [47]. In 2007, further investigation by Dr. Brinkmann's group concluded NETosis was a cell death pathway [48, 49]. This process was later described in other granulocyte lineage cells [43, 50], but was not found in monocites or basophiles [29, 43, 49]. Taking these findings into account, the term ETosis was born and describes the process of cell death that leads to extracellular traps formation [51], using NETosis specifically when these ETs are produced by neutrophils (NETs) [52]. 

After the stimulation of a population of neutrophils through various means [9]: LPS, IL-8, interferon gamma, PMA, monosodium urate cristals [43], and microorganisms [10], only a portion of the population will generate NETs. Among the aforementioned stimuli, microorganisms or PMA are the most potent agents inducing NETosis in approximately 30% of the population, suggesting the importance of different activation pathways in this process [48]. EM analyses have shed light on the chain of events that occurs during NET formation. First of all, after activation, neutrophils present a flattening of their cellular structure with the visualization of multiple cytoplasmatic vacuoles. Secondly, the distinction between euchromatin and heterochromatin is lost as well as their characteristic nuclear lobulations and a space between the inner and outer nuclear membrane is formed. Thirdly, the nuclei increased its size to occupy most of the cytoplasm and the integrity of the nuclear and granular membranes is lost allowing the components of NETs to mix [49]. All this process is carried out while the cytoplasmatic membrane remains unharmed. In the last stage, neutrophils die releasing the ET and expressing death cell indicators such as phosphatidylserine [29]. 

## 6. Biochemical Events

NET formation requires two events: the production of ROS and chromatin unfolding [53]. ROS production is performed by the enzyme NOX2 [48] and is responsible for the oxidative burst that will ultimately kill the phagocytized organism in the phagolysosome [52]. The intracellular steps leading ROS to create NETs are not completely understood, nonetheless it is known that the protein kinase C (PKC) pathway is important given the fact that PMA (an activator of the former) is one of the most potent inducers of NET formation known to date [48]. The activation of the PKC pathway leads to the assembly of the NOX2 complex in the phagosome membrane and the electron transport inside it generates superoxide anions (O_2_^−^). At the same time, the increase in the negative charge generated in the process creates a favorable gradient for the hydrogen ions (H^+^) to enter the phagosome and mix with superoxide anion generating hydrogen peroxide (H_2_O_2_). This compound, unlike other ROS, is electroneutral and can diffuse back to the cytoplasm [52] where it turns the cellular balance toward generalized activation through the oxidation of the tyrosine phosphatase superfamily in a specific cysteine residue highly conserved in this protein superfamily leaving such enzymes inactive in a reversible or irreversible fashion depending on the degree of oxidation [54]. Other enzymes that are target of peroxidation through cysteine residues are caspases, which therefore block apoptosis [55]. The evidence of the importance of ROS in the generation of NETosis is based on the following evidence.

1. H_2_O_2_ is a potent inducer of NET generation at physiologic concentrations [49].
2. The inhibition of NOX2 blocks the their production [48].
3. Adding catalase to neutrophil cultures by converting H_2_O_2_ to water blocks NET formation [48].
4. In patients with chronic granulomatous disease (CGD), a disease caused by the deficiency of any of the NOX2 components, there is an immunosuppressive syndrome leading to life-threatening infections and to the absence of NET formation under EM [56]. Additionally, in order to reestablish NOX2 activity, gene therapy restores NET formation efficiently, which clears the infection [25].

A word of caution—recently a ROS-independent pathway was described in the NET formation [34]. This finding has been recently retracted by the authors after they found their culture conditions generated spontaneous neutrophil activation [57]. To date, ROS are sine qua non for NET formation. 

The second process is the decondensation of chromatin, a process which is only partially understood. Studies using a cell-free medium containing low-density supernatants (LDS) of neutrophils (cytoplasm and granules) produced nuclear decondensation in different cellular lines. Further separation of LDS revealed that neutrophil elastase stored in the azurophilic granules was the etiological agent responsible for this phenomenon [58]; this enzyme is translocated to the nucleus after neutrophil activation and degrades histones especially H4, with the nuclear changes and chromatin decondensation being directly proportional to the level of H4 degradation [58]. Myeloperoxidase, another enzyme stored within these granules, does not produce histone degradation per se, although it enhances NE degradation [58] and is necessary for NET formation [59]. Another important step in this process is the citrullination of histones through peptidyl arginine deiminase 4 (PD4), a calcium-dependent enzyme present in neutrophils [29, 60], and the only one within this ensuing family with a nuclear localization dominium allowing it to work at such level [61]. The histone citrullination prevents histone methylation and further transcription and allows chromatin to decondense. In experimental murine models lacking PD4, NETs are absent upon stimulation and so are the nuclear changes innate to this process [59, 62]. 

## 7. NETosis and Infection

The microbicide effects of NETs have been confirmed in several animal and human models with fungal, parasitic, bacterial, and viral infections [37] (Table 3), although it is somewhat more difficult to use murine models taking into account the lower percentage of neutrophils in blood in mice compared with humans [26]. As mentioned previously its function depends on its scaffold of DNA and histone as demonstrated by the loss of microbicidal capacities upon exposure to DNA-degrading enzymes [9]. Histones are not only important because of their structural properties but also due to their intrinsic bactericidal character recognized for almost half a century [63]. Another important property is the capacity to snare organisms within it allowing the action of the different granular substance attached [9, 37]. Therefore, it is not surprising that these microorganisms developed strategies to survive NETs, these mechanisms are better exemplified in gram-positives where DNAse, nuclease [64, 65], and capsule generation [66] allow bacteria to survive and escape these NETs. *In vitro* models using human neutrophils and human inmmunodeficiency virus (HIV) have shown not only that human neutrophils form NETs upon exposure to HIV but also that the virus generates high levels of interleukin-10 via c-type lectin CD209 dependent production by dendritic cells in order to prevent this event [37]. It is important to underline how in some special conditions like sepsis, high LPS concentration can induce the production of NETs in the intravascular space thanks to atypical platelet activation through toll-like receptors (TLR) 4 [22]. These NETs are capable of withstanding pulmonary, capillary, and hepatic sinusoidal flow velocity locations where NETs are predominantly formed. They also facilitate the colocalization of platelets and neutrophils so the former can help activate the latter in an attempt to depurate the bacteremia at the expense of further endothelial damage [22, 67]. This phenomenon requires higher LPS levels than the ones eliciting a maximum response in neutrophils by direct action, which is observed *in vivo* only with high level bacteremia [22]. The liberation of NETs within the circulation unmask the dark side of histones that generate and increase the thrombin levels [68] producing a procoagulant and cytotoxic environment to the endothelial cell, which is ultimately associated in mouse and baboon models of sepsis with an increase in mortality [69, 70]. Finally, the intracellular pathways that set the fate of the neutrophils are unknown, but if bacteria are accessible to neutrophils, it initiates phagocytosis soon after activation, otherwise the final fate would probably be NETosis [71].

## 8. NETs in Noninfectious Disease

NETs have been observed in noninfectious models of reproductive medicine, from fecundation [72], where NETs are formed in order to trap the male gamete and avoid fecundation and preeclampsia where syncytiotrophoblast particles possess the ability to induce NET formation [73] and generate placental hypoxia [32]. 

In autoimmune diseases, as in small-vessel vasculitis, the neutrophil-associated antibodies (ANCAS) can induce NET formation and glomerular deposition in a similar way to PMA [42]. In SLE, neutrophils have gained relevance, specifically a subgroup called low-density neutrophils (LDN), which possess an intrinsic proinflammatory phenotype including type 1 interferon and TNF alfa secretion with reduced phagocytic capacities and an increased NET formation [38]. These NETs have been observed in renal and cutaneous tissue of affected individuals and have been shown to cause direct cytotoxicity to the endothelial cell [41]. Likewise, deficient NET degradation has been observed in lupus nephritis patients [39]. Finally, since these LDN can increase the production of type 1 interferon by plasmacytoid dendritic cells and antigen presentation to B lymphocytes favoring antibody production and generate the interferon signature, these LDN are recognized as important cells in the initiation of autoimmunity and its posterior maintenance [74]. 

## 9. Mitochondrial “NET-Like” Structures

A new structure similar to ETs was described by Yusefi et al. in eosinophils [33]. These structures are composed of mitochondrial DNA and did not imply nuclear destruction or cell death. Neutrophils showed the capacity to perform mitochondrial NET-like structures as well and, given the eminently anaerobic metabolism of neutrophils, the reduced number of mitochondria they possess, and the absence of granular proteins attached to mitochondrial DNA, it has been proposed that mitochondrial NET like structures serve as a mechanism to avoid apoptosis or as an amplification device using DNA through TLR 9 to produce cellular activation in surrounding cells [75, 76]. Whether or not these mitochondrial structures should be considered ET is still a matter of debate and more evidence is required before drawing any conclusion.

## 10. Final Remarks

NETosis is a cell death pathway whose principal consequence is ET formation. These ETs are important in order to control extracellular infections and constitute a way to concentrate the microbicide armamentarium limiting collateral tissue damage. Under special circumstances, such as sepsis and some autoimmune diseases, excessive formation or deficient degradation can lead to organ damage and perpetuation of the autoimmune response. 

## Figures and Tables

**Table 1:** Cell death.

Morphological criteria: dead cell	Point of no return: dying cell
Loss of plasma membrane integrity	Massive activation of caspases (present also in differentiation and activation of cells)
Cell fragmentation	Mitochondrial transmembrane permeabilization
Engulfment by adjacent cells	Mitochondrial membrane permeabilization (with leak of catabolic enzymes or enzymatic activators)
	Exposure of phosphatidylserine in the outer membrane (could be a transient process, e.g., T-cell activation)

**Table 2:** NET components.

Nuclear components [9]	DNA
	Histones
Granular components [10]	
(i) Primary granules	Myeloperoxidase, cathepsin G, neutrophil elastase
(ii) Secondary granules	Lactoferrin, pentraxin 3 [11]
(iii) Tertiary granules	Gelatinase, peptidoglycan-binding protein [12]
Cytoplasmatic components:	Calprotectin, catalase [13]

**Table 3:** NETosis models.

Animals	
(i) Feline leukemia virus [20]	
(ii) Candida murine infection in chronic granulomatous	
(iii) Disease [21]	
(iv) hominid sepsis [22]	
(v) bovine mastitis [23]	
Humans	
(i) Candida albicans [24]	
(ii) Aspergillus nidulans [25], A fumigatus [26]	
(iii) Leishmaniasis [27]	
(iv) Shigella flexneri [28]	
(v) Staphylococcus aureus [9]	
(vi) Salmonella typhimurium [9]	
(vii) Group A Streptococcus [29]	
(viii) Gingivitis [30, 31]	
(ix) Preeclampsia [32]	
(x) Chron's disease [33]	
(xi) Cystic fibrosiss [34]	
(xii) Tuberculosis [35]	
(xiii) Malaria [36]	
(xiv) Human immunodeficiency virus [37]	
(xv) Chronic granulomatous disease [25]	
(xvi) Systemic lupus erythematosus [38–41]	
(xvii) Small-vessel vasculitis [42]	
(xviii) Monosodium urate cristals [43]	

## References

[1] Mantovani A, Cassatella MA, Costantini C, Jaillon S (2011). Neutrophils in the activation and regulation of innate and adaptive immunity. *Nature Reviews Immunology*.

[2] Abul A, Abul A (2008). Inmunidad innata. *Inmunologia Celular Y Molecular*.

[3] Kumar V, Sharma A (2010). Neutrophils: cinderella of innate immune system. *International Immunopharmacology*.

[4] Cabrini M, Nahmod K, Geffner J (2010). New insights into the mechanisms controlling neutrophil survival. *Current Opinion in Hematology*.

[5] Colotta F, Re F, Polentarutti N, Sozzani S, Mantovani A (1992). Modulation of granulocyte survival and programmed cell death by cytokines and bacterial products. *Blood*.

[6] Serhan CN, Chiang N, Van Dyke TE (2008). Resolving inflammation: dual anti-inflammatory and pro-resolution lipid mediators. *Nature Reviews Immunology*.

[7] Romano M, Serhan CN (1992). Lipoxin generation by permeabilized human platelets. *Biochemistry*.

[8] Nathan C (2006). Neutrophils and immunity: challenges and opportunities. *Nature Reviews Immunology*.

[9] Brinkmann V, Reichard U, Goosmann C (2004). Neutrophil extracellular traps kill bacteria. *Science*.

[10] Wartha F, Beiter K, Normark S, Henriques-Normark B (2007). Neutrophil extracellular traps: casting the NET over pathogenesis. *Current Opinion in Microbiology*.

[11] Jaillon S, Peri G, Delneste Y (2007). The humoral pattern recognition receptor PTX3 is stored in neutrophil granules and localizes in extracellular traps. *Journal of Experimental Medicine*.

[12] Cho JH, Fraser IP, Fukase K (2005). Human peptidoglycan recognition protein S is an effector of neutrophil-mediated innate immunity. *Blood*.

[13] Bianchi M, Niemiec MJ, Siler U, Urban CF, Reichenbach J (2011). Restoration of anti-Aspergillus defense by neutrophil extracellular traps in human chronic granulomatous disease after gene therapy is calprotectin-dependent. *Journal of Allergy and Clinical Immunology*.

[14] Kerr JF, Wyllie AH, Currie AR (1972). Apoptosis: a basic biological phenomenon with wide-ranging implications in tissue kinetics. *British Journal of Cancer*.

[15] Kroemer G, El-Deiry WS, Golstein P (2005). Classification of cell death: recommendations of the Nomenclature Committee on Cell Death. *Cell Death and Differentiation*.

[16] Kroemer G, Galluzzi L, Vandenabeele P (2009). Classification of cell death: recommendations of the Nomenclature Committee on Cell Death 2009. *Cell Death and Differentiation*.

[17] Galluzzi L, Vitale I, Abrams JM (2012). Molecular definitions of cell death subroutines: recommendations of the Nomenclature Committee on Cell Death 2012. *Cell Death and Differentiation*.

[18] Rubinsztein DC, Mariño G, Kroemer G (2011). Autophagy and aging. *Cell*.

[19] Eisenberg-Lerner A, Bialik S, Simon HU, Kimchi A (2009). Life and death partners: apoptosis, autophagy and the cross-talk between them. *Cell Death and Differentiation*.

[20] Wardini AB, Guimarães-Costa AB, Nascimento MTC (2010). Characterization of neutrophil extracellular traps in cats naturally infected with feline leukemia virus. *Journal of General Virology*.

[21] Ermert D, Urban CF, Laube B, Goosmann C, Zychlinsky A, Brinkmann V (2009). Mouse neutrophil extracellular traps in microbial infections. *Journal of Innate Immunity*.

[22] Clark SR, Ma AC, Tavener SA (2007). Platelet TLR4 activates neutrophil extracellular traps to ensnare bacteria in septic blood. *Nature Medicine*.

[23] Lippolis JD, Reinhardt TA, Goff JP, Horst RL (2006). Neutrophil extracellular trap formation by bovine neutrophils is not inhibited by milk. *Veterinary Immunology and Immunopathology*.

[24] Urban CF, Ermert D, Schmid M (2009). Neutrophil extracellular traps contain calprotectin, a cytosolic protein complex involved in host defense against Candida albicans. *PLoS Pathogens*.

[25] Bianchi M, Hakkim A, Brinkmann V (2009). Restoration of NET formation by gene therapy in CGD controls aspergillosis. *Blood*.

[26] Bruns S, Kniemeyer O, Hasenberg M (2010). Production of extracellular traps against Aspergillus fumigatus in vitro and in infected lung tissue is dependent on invading neutrophils and influenced by hydrophobin RodA. *PLoS Pathogens*.

[27] Guimarães-Costa AB, Nascimento MTC, Froment GS (2009). Leishmania amazonensis promastigotes induce and are killed by neutrophil extracellular traps. *Proceedings of the National Academy of Sciences of the United States of America*.

[28] Medina E (2009). Neutrophil extracellular traps: a strategic tactic to defeat pathogens with potential consequences for the host. *Journal of Innate Immunity*.

[29] Remijsen Q, Kuijpers TW, Wirawan E, Lippens S, Vandenabeele P, Vanden Berghe T (2011). Dying for a cause: NETosis, mechanisms behind an antimicrobial cell death modality. *Cell Death and Differentiation*.

[30] Krautgartner WD, Klappacher M, Hannig M (2010). Fibrin mimics neutrophil extracellular traps in SEM. *Ultrastructural Pathology*.

[31] Vitkov L, Klappacher M, Hannig M, Krautgartner WD (2010). Neutrophil fate in gingival crevicular fluid. *Ultrastructural Pathology*.

[32] Gupta AK, Hasler P, Holzgreve W, Hahn S (2007). Neutrophil NETs: a novel contributor to preeclampsia-associated placental hypoxia?. *Seminars in Immunopathology*.

[33] Yousefi S, Gold JA, Andina N (2008). Catapult-like release of mitochondrial DNA by eosinophils contributes to antibacterial defense. *Nature Medicine*.

[34] Marcos V, Zhou Z, Yildirim AO (2010). CXCR2 mediates NADPH oxidase-independent neutrophil extracellular trap formation in cystic fibrosis airway inflammation. *Nature Medicine*.

[35] Ramos-Kichik V, Mondragón-Flores R, Mondragón-Castelán M (2009). Neutrophil extracellular traps are induced by Mycobacterium tuberculosis. *Tuberculosis*.

[36] Baker VS, Imade GE, Molta NB (2008). Cytokine-associated neutrophil extracellular traps and antinuclear antibodies in Plasmodium falciparum infected children under six years of age. *Malaria Journal*.

[37] Saitoh T, Komano J, Saitoh Y (2012). Neutrophil extracellular traps mediate a host defense response to human. *Cell Host & Microbe*.

[38] Denny MF, Yalavarthi S, Zhao W (2010). A distinct subset of proinflammatory neutrophils isolated from patients with systemic lupus erythematosus induces vascular damage and synthesizes type I IFNs. *Journal of Immunology*.

[39] Hakkim A, Fürnrohr BG, Amann K (2010). Impairment of neutrophil extracellular trap degradation is associated with lupus nephritis. *Proceedings of the National Academy of Sciences of the United States of America*.

[40] Obermoser G, Pascual V (2010). The interferon-*α* signature of systemic lupus erythematosus. *Lupus*.

[41] Villanueva E, Yalavarthi S, Berthier CC (2011). Netting neutrophils induce endothelial damage, infiltrate tissues, and expose immunostimulatory molecules in systemic lupus erythematosus. *Journal of Immunology*.

[42] Kessenbrock K, Krumbholz M, Schönermarck U (2009). Netting neutrophils in autoimmune small-vessel vasculitis. *Nature Medicine*.

[43] Schorn C, Janko C, Latzko M, Chaurio R, Schett G, Herrmann M (2012). Monosodium urate crystals induce extracellular DNA traps in neutrophils. *Frontiers in Immunology*.

[44] Simon HU (2003). Neutrophil apoptosis pathways and their modifications in inflammation. *Immunological Reviews*.

[45] Mihalache CC, Yousefi S, Conus S, Villiger PM, Schneider EM, Simon HU (2011). Inflammation-associated autophagy-related programmed necrotic death of human neutrophils characterized by organelle fusion events. *Journal of Immunology*.

[46] Brinkmann V, Laube B, Abu Abed U, Goosmann C, Zychlinsky A (2010). Neutrophil extracellular traps: how to generate and visualize them. *Journal of Visualized Experiments*.

[47] Lee WL, Grinstein S (2004). Immunology. The tangled webs that neutrophils weave. *Science*.

[48] Brinkmann V, Zychlinsky A (2007). Beneficial suicide: why neutrophils die to make NETs. *Nature Reviews Microbiology*.

[49] Fuchs TA, Abed U, Goosmann C (2007). Novel cell death program leads to neutrophil extracellular traps. *Journal of Cell Biology*.

[50] von Köckritz-Blickwede M, Goldmann O, Thulin P (2008). Phagocytosis-independent antimicrobial activity of mast cells by means of extracellular trap formation. *Blood*.

[51] Wartha F, Henriques-Normark B (2008). ETosis: a novel cell death pathway. *Science Signaling*.

[52] Steinberg BE, Grinstein S (2007). Unconventional roles of the NADPH oxidase: signaling, ion homeostasis, and cell death. *Science’s STKE*.

[53] Amulic B, Hayes G (2011). Neutrophil extracellular traps. *Current Biology*.

[54] Tonks NK (2005). Redox redux: revisiting PTPs and the control of cell signaling. *Cell*.

[55] Fadeel B, Åhlin A, Henter JI, Orrenius S, Hampton MB (1998). Involvement of caspases in neutrophil apoptosis: regulation by reactive oxygen species. *Blood*.

[56] Babior BM (2004). NADPH oxidase. *Current Opinion in Immunology*.

[57] Marcos V, Zhou Z, Yildirim AO (2011). CXCR2 mediates NADPH oxidase-independent neutrophil extracellular trap formation in cystic fibrosis airway inflammation. *Nature Medicine*.

[58] Papayannopoulos V, Metzler KD, Hakkim A, Zychlinsky A (2010). Neutrophil elastase and myeloperoxidase regulate the formation of neutrophil extracellular traps. *Journal of Cell Biology*.

[59] Metzler KD, Fuchs TA, Nauseef WM (2011). Myeloperoxidase is required for neutrophil extracellular trap formation: implications for innate immunity. *Blood*.

[60] Wang Y, Li M, Stadler S (2009). Histone hypercitrullination mediates chromatin decondensation and neutrophil extracellular trap formation. *Journal of Cell Biology*.

[61] Mastronardi FG, Wood DD, Mei J (2006). Increased citrullination of histone H3 in multiple sclerosis brain and animal models of demyelination: a role for tumor necrosis factor-induced peptidylarginine deiminase 4 translocation. *Journal of Neuroscience*.

[62] Li P, Li M, Lindberg MR, Kennett MJ, Xiong N, Wang Y (2010). PAD4 is essential for antibacterial innate immunity mediated by neutrophil extracellular traps. *Journal of Experimental Medicine*.

[63] Hirsch JG (1958). Bactericidal action of histone. *The Journal of Experimental Medicine*.

[64] Beiter K, Wartha F, Albiger B, Normark S, Zychlinsky A, Henriques-Normark B (2006). An endonuclease allows Streptococcus pneumoniae to escape from neutrophil extracellular traps. *Current Biology*.

[65] Buchanan JT, Simpson AJ, Aziz RK (2006). DNase expression allows the pathogen group A Streptococcus to escape killing in neutrophil extracellular traps. *Current Biology*.

[66] Wartha F, Beiter K, Albiger B (2007). Capsule and D-alanylated lipoteichoic acids protect Streptococcus pneumoniae against neutrophil extracellular traps. *Cellular Microbiology*.

[67] Ma AC, Kubes P (2008). Platelets, neutrophils, and neutrophil extracellular traps (NETs) in sepsis. *Journal of Thrombosis and Haemostasis*.

[68] Ammollo CT, Semeraro F, Xu J, Esmon NL, Esmon CT (2011). Extracellular histones increase plasma thrombin generation by impairing thrombomodulin-dependent protein C activation. *Journal of Thrombosis and Haemostasis*.

[69] Xu J, Zhang X, Pelayo R (2009). Extracellular histones are major mediators of death in sepsis. *Nature Medicine*.

[70] Chaput C, Zychlinsky A (2009). Sepsis: the dark side of histones. *Nature Medicine*.

[71] Papayannopoulos V, Zychlinsky A (2009). NETs: a new strategy for using old weapons. *Trends in Immunology*.

[72] Alghamdi AS, Foster DN (2005). Seminal DNase frees spermatozoa entangled in neutrophil extracellular traps. *Biology of Reproduction*.

[73] Gupta AK, Hasler P, Holzgreve W, Gebhardt S, Hahn S (2005). Induction of neutrophil extracellular DNA lattices by placental microparticles and IL-8 and their presence in preeclampsia. *Human Immunology*.

[74] Bosch X (2011). Systemic lupus erythematosus and the neutrophil. *The New England Journal of Medicine*.

[75] Lögters T, Margraf S, Altrichter J (2009). The clinical value of neutrophil extracellular traps. *Medical Microbiology and Immunology*.

[76] Melino G (2001). The Sirens’ song. *Nature*.

